# Dynamic remodeling of septin structures fine-tunes myogenic differentiation

**DOI:** 10.1016/j.isci.2024.110630

**Published:** 2024-07-31

**Authors:** Vladimir Ugorets, Paul-Lennard Mendez, Dmitrii Zagrebin, Giulia Russo, Yannic Kerkhoff, Georgios Kotsaris, Jerome Jatzlau, Sigmar Stricker, Petra Knaus

**Affiliations:** 1 Freie Universität Berlin, Institute of Chemistry and Biochemistry, Signal Transduction Group, 14195 Berlin, Germany; 2 Leibniz-Forschungsinstitut für Molekulare Pharmakologie (FMP), 13125 Berlin, Germany; 3 Freie Universität Berlin, Institute of Chemistry and Biochemistry, Bionanointerfaces Group, 14195 Berlin, Germany; 4 Berlin-Brandenburg School for Regenerative Therapies, Charité - Universitätsmedizin Berlin, 13353 Berlin, Germany; 5 Freie Universität Berlin, Institute of Chemistry and Biochemistry, Musculoskeletal Development and Regeneration Group, 14195 Berlin, Germany; 6 Max Planck Institute for Molecular Genetics, IMPRS-Biology and Computation, 14195 Berlin, Germany

**Keywords:** Cell biology, Organizational aspects of cell biology, Developmental biology

## Abstract

Controlled myogenic differentiation is integral to the development, maintenance and repair of skeletal muscle, necessitating precise regulation of myogenic progenitors and resident stem cells. The transformation of proliferative muscle progenitors into multinuclear syncytia involves intricate cellular processes driven by cytoskeletal reorganization. While actin and microtubles have been extensively studied, we illuminate the role of septins, an essential yet still often overlooked cytoskeletal component, in myoblast architecture. Notably, Septin9 emerges as a critical regulator of myoblast differentiation during the initial commitment phase. Knock-down of Septin9 in C2C12 cells and primary mouse myoblasts accelerates the transition from proliferation to committed progenitor transcriptional programs. Furthermore, we unveil significant reorganization and downregulation of Septin9 during myogenic differentiation. Collectively, we propose that filmamentous septin structures and their orchestrated reorganization in myoblasts are part of a temporal regulatory mechanism governing the differentiation of myogenic progenitors. This study sheds light on the dynamic interplay between cytoskeletal components underlying controlled myogenic differentiation.

## Highlights

• Septins are an integral part of myoblast cytoskeleton
• Septin architecture reorganizes substantially during differentiation
• Septin9 expression is downregulated during differentiation
• Depleting Septin9 accelerates transition to the committed progenitor state

## Graphical abstract

**Figure:**
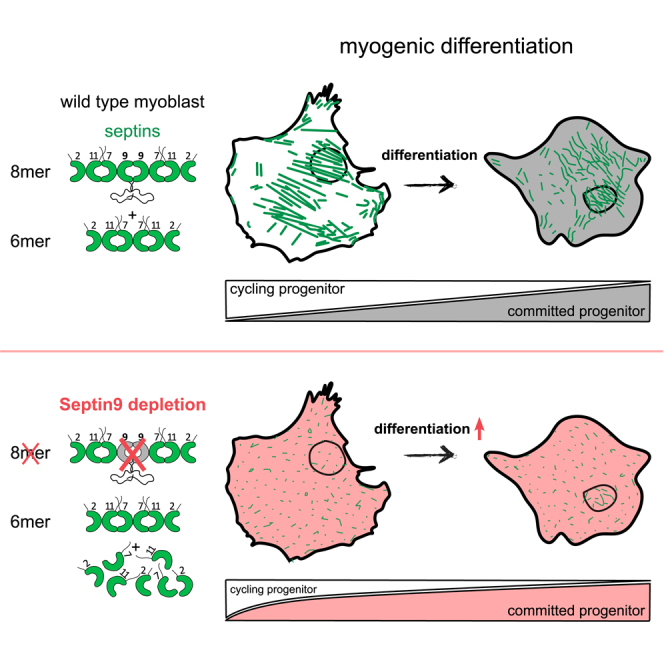

## Introduction

Skeletal muscle is a dynamic tissue making up about 40% of the body and is essential for many vital functions.^1^ It forms during development in the process of myogenesis, that also occurs in adult organisms during muscle regeneration. During these processes, myogenic precursor cells proliferate, migrate to the location of muscle formation, differentiate into mature muscle cells and fuse to form muscle fibers.^2^ Myogenic differentiation induces characteristic changes in gene expression and cell morphology in proliferating myogenic precursor cells (myoblasts) that transition toward contractile fibers, first transforming into fusion-competent progenitors (myocytes) and eventually fusing to give rise to new myotubes or enlarging existing myotubes.^3^^,^^4^ These intricate cellular changes coincide with adaptations in the organization of cytoskeletal proteins, including actin^5^^,^^6^^,^^7^ and tubulin.^8^^,^^9^

Among the cytoskeletal players, septins have gained increasing attention. Septins, a family of GTP binding proteins, have begun to emerge in the context of myogenesis.^10^^,^^11^^,^^12^^,^^13^^,^^14^ Classified into four subgroups (Septin2-Septin6-Septin7-Septin9), they primarily differ in their N- and C-terminal extensions.^15^ These proteins assemble into palindromic hetero-octamers (referred to as protomer), composed of one subunit from each subgroup. By lateral interactions, septins build higher-order structures like bundles, rings and networks. Their capacity to bind the plasma membrane, actin or microtubules defines their functions as lateral diffusion barriers or compartmentalizing scaffolds.^16^^,^^17^^,^^18^ Septins have been implicated in diverse cellular functions, including cytokinesis,^19^ cell mobility^20^ and contractility,^21^ plasma membrane rigidity,^22^^,^^23^ cell shape determination^24^^,^^25^^,^^26^^,^^27^ and mechanotransduction.^28^^,^^29^

This study focuses on Septin9, a centrally positioned paralog within the protomer,^30^ the embryonic depletion of which is lethal.^31^ The distinct N-terminal extensions of Septin9 enable interactions with both actin and microtubules,^17^ potentially bridging actin to the plasma membrane.^32^ However, understanding the expression, organization, and functions of septins in muscle formation remains limited. While human septin transcripts have been detected in sleletal muscle and heart tissue,^33^ the role of specific septins in skeletal muscle has only recently been explored. For instance, Septin7 was found essential for cardiac and somatic myofibril organization in zebrafish,^34^ murine myoblast proliferation, skeletal muscle architecture, and function.^11^ This investigation aims to shed light on the involvement of Septin9 in the context of myogenic differentiation, elucidating its functions and contributions in this dynamic process.

## Results

### Expression of septins during muscle regeneration and *in vitro* myogenic differentiation

**Figure 1:**
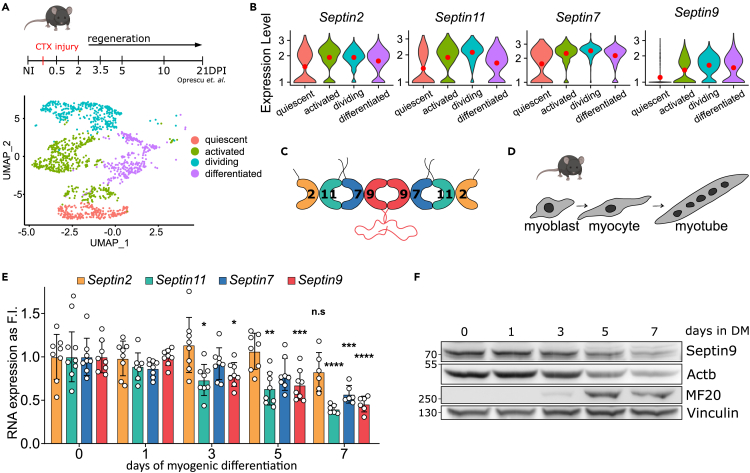
Expression of septins during muscle regeneration and *in vitro* myogenic differentiation (A and B) Single cell RNA sequencing data of non-injured tibialis anterior muscle and additional six time points following cardiotoxin injury (CTX).^35^ (A) UMAP visualization of the sub-cluster MuSC in regenerating musculature colored by identified cell populations within the sub-clusters. MuSC labeled cells were extracted from annotated single cell dataset available from GSE138826. (B) Violin plots representing gene expression profiles of *Septin2, 7, 9* and *11* in MuSC subclusters. (C) Potential core myogenic septin protomer in non-qiescent MuSCs. (D) Schematic representation of murine *in vitro* myogenic differentiation. (E) mRNA expression levels of *Septin2*, *7*, *9* and *11* during C2C12 differentiation. Septins are color coded according to the subfamily, as in (C). (F) Protein levels of Septin9 during C2C12 differentiation. Data represent mean ± standard deviation (SD), ∗*p* < 0.05, ∗∗*p* < 0.01, ∗∗∗*p* < 0.001, ∗∗∗∗*p* < 0.0001, n.s – not significant from one way ANOVA followed by Dunnett’s multiple comparison test. See also Figures S1 and S2.

To identify the septins that are expressed in myogenic progenitors, we analyzed single-cell RNA-sequencing (scRNA-seq) data from muscle stem cells (MuSCs) of regenerating tibialis anterior (TA) muscle, spanning 0.5, 2, 3.5, 5, 10 and 21 days post cardiotoxin injury.^35^ Unsupervised clustering revealed four sub-clusters including quiescent, activated, dividing, and differentiated MuSCs that were identified according to expressed marker genes (see STAR Methods) (Figures 1A and S1A–S1C). This revealed *Septin2*, *7*, *10* and *11* to be expressed in all MuSC populations, while *Septin8* and *Septin9* were exempt from quiescent cells (Figures 1B and S1D). *Septin6* specifically marked proliferating cell population and *Septin4* marked the differentiated population (Figure S1D). The most highly expressed septins within every septin homology group were *Septin2, 7* and *11* for quiescent MuSCs and *Septin2, 7, 9* and *11* for all other myogenic sub-clusters, thus providing an insight into the potential protomere assembly during myogenic lineage progression (Figures 1B and 1C). Organizing the gene expression according to the regeneration time points, it is apparent that expression of core Septin paralogues (2, 7, 9, 11) was induced concomitant with the onset of muscle regeneration while it steadily declined with time and is majorly downregulated by day 21 when the regeneration is assumed to be nearly complete. Interestingly, only *Septin9* expression completely diminished by day 21. Next, we confirmed the expression of septins (except *Septin1, 3*, 12 and *14*, very low or not detectable) during 7 days of *in vitro* myogenic differentiation in C2C12 cells (Figures 1E, S2A, and S2B) and primary myoblasts (5 days of differentiation) (Figures S2C–S2F). These experiments consistently demonstrated the downregulation of *Septin9* from day 3 onwards (Figures 1E and S2C). Additionally, we observed the downregulation of *Septin6* and *7* and upregulation of *Septin4* in both, C2C12 cells and primary myoblasts (Figures 1E and S2A–S2E). To confirm the transcriptional data, we analyzed Septin9 protein expression via western blot analysis and similarly observed the reduction of Septin9 during myogenic differentiation in C2C12 cells and primary myoblasts (Figures 1F and S2F). In summary, our finding reveal a global downregulation of multiple ubiquitous septins during myogenic differentiation, with the exception of Septin4, which displays distinct upregulation. Furthermore, quiescent MuSCs and proliferating myoblasts may potentially rely on different sets of septin protomeres, with Septin9 not present in quiescent cells, and being downregulated during regenerative myogenesis and *in vitro* myogenic differentiation.

### Septin9 depletion promotes transition from dividing toward differentiating progenitors on mRNA level

**Figure 2:**
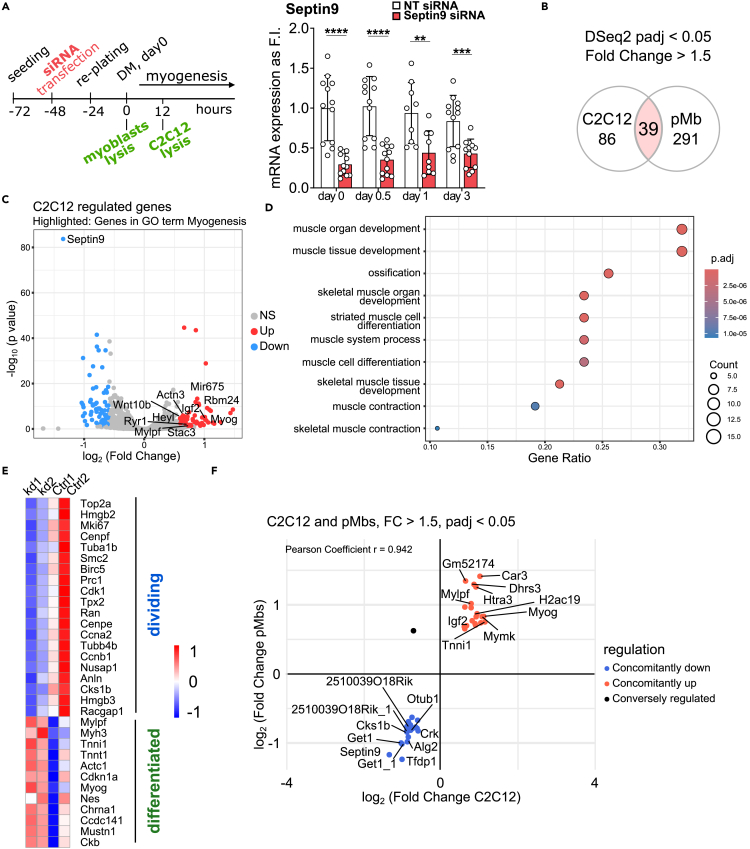
Septin9 depletion induces transition from cycling toward committed C2C12 cells (A) Schematic representation of the experimental setup. Total RNA was isolated from proliferating myoblasts 48 h after Septin9 knockdown and from 12 h differentiating C2C12 cells (60 h after knockdown). Septin9 knockdown efficiency validation over 3 days of *in vitro* myogenesis via qRT-PCR in C2C12 cells. (B) Venn diagram comparing DEGs from C2C12 cells and primary myoblasts calculated using DESeq2 (adjusted *p* value <0.05; −0.585 ≤ log2FC ≤ 0.585). (C) Volcano plot of DE genes (adjusted *p* value < 0.05; −0.585 ≤ log2FC ≤ 0.585) of C2C12 cells transfected with either non-targeting siRNA (control) or Septin9 siRNA after 12 h of myogenic differentiation, genes corresponding to Gene Ontology term “Myogenesis” are highlighted. (D) GO enrichment analysis of genes upregulated in Septin9-deficient C2C12 cells. (E) Heatmap depicting marker genes for dividing and differentiated MuSCs defined by scRNAseq data depicted in Figure 1. Genes are filtered for significant regulation in C2C12 (adjusted *p* value < 0.05). Data represent mean ± standard deviation (SD), ∗*p* < 0.05, ∗∗*p* < 0.01, ∗∗∗*p* < 0.001, ∗∗∗∗*p* < 0.0001 from two-sided unpaired t test. (F) Volcano plot depicting 39 common DEGs between C2C12 and primary myoblasts, calculated using DESeq2 (adjusted *p* value <0.05; −0.585 ≤ log2FC ≤ 0.585). See also Figure S3.

Septin9 plays a crucial role in determining the subcellular localization of the septin cytoskeleton and in regulating cytoarchitecture.^32^^,^^36^ To understand the implications of Septin9 downregulation, we investigated whether its reduction in expression merely eliminates a vestigial cytoskeletal component no longer required during late myogenic differentiation or if it potentially serves an instructive role in governing the progression through the process. Thus, we employed a siRNA-based approach to deplete Septin9 (Figure 2A). We conducted RNA sequencing analysis of Septin9 depleted C2C12 cells undergoing differentiation (after 12 h of differentiation, Figure 2) and Septin9 depleted primary myoblasts in a proliferative state (Figure S3). Differential expression analysis revealed 125 differentially expressed genes (DEGs) in Septin9-deficient C2C12 cells, with 71 genes upregulated and 54 genes downregulated when compared to control cells transfected with non-targeting siRNA (Figures 2B and 2C). Similarly, we identified 330 DEGs in Septin9-deficient primary myoblasts comprising 285 upregulated and 45 downregulated genes (Figures 2B and S3B). Upon conducting gene ontology (GO) annotation analysis of DEGs in Septin9-deficient C2C12 cells according to biological process, we found that upregulated genes were associated with the terms such as ‘muscle organ development’, ‘skeletal muscle cell differentiation’, ‘muscle contraction’, and ‘myotube differentiation’ (Figure 2D). Similar results were observed in Septin9-deficient proliferating myoblasts (Figure S3C). To deepen our understanding of Septin9’s role in myogenic differentiation,we compared the DEG profiles of Septin9-deficient C2C12 cells (Figure 2E) and primary moyblasts (Figure S3D) with those of proliferating and differentiated muscle stem cells (MuSCs), previously defined by single-cell RNA-sequencing^3^ (Figure 1A). In both Septin9-deficient C2C12 cells and primary myoblasts, we observed an upregulation of genes associated with the ‘differentiated’ cluster, along with a mild downregulation of genes related to cell proliferation. This suggests a premature transition of the cycling progenitor population toward committed progenitors. Furthermore, we analyzed the set of 39 DEGs that was shared between Septin9-deficient C2C12 and primary myoblasts (Figure 2F). Except for Acta2, 38 of the common DEGs demonstrated simultaneous up- or downregulation in both cell types following Septin9 depletion. Upregulated genes include marker genes for late stages of myogenic differentiation such as Myogenin (*Myog*), Myomaker (*Mymk*), Insulin like growth factor 2 (*Igf2*) and slow Troponin1 (*Tnni1*) among others. Together, the analysis of DEGs upon Septin9 depletion in C2C12 cells and primary myoblasts suggests a premature transition from proliferating to differentiating myogenic progenitors during myogenic differentiation.

### Septin9 depletion accelerates myogenic differentiation in C2C12 cells

**Figure 3:**
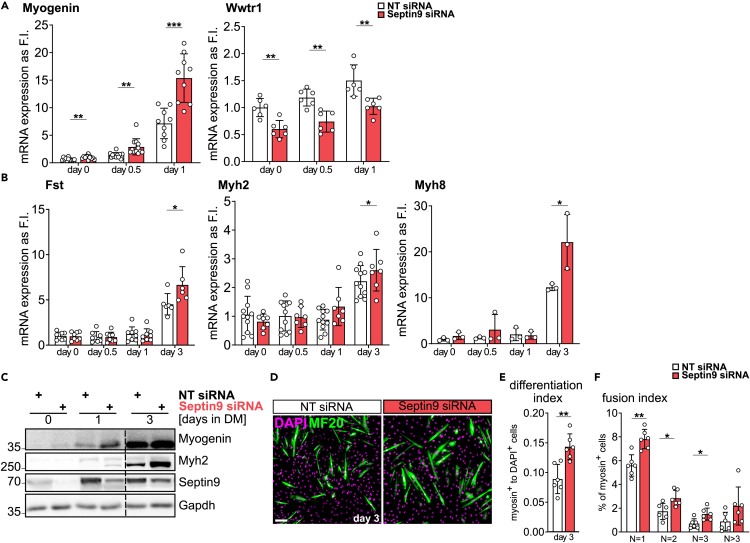
Septin9 depletion accelerates myogenic differentiation in C2C12 cells (A) The qRT-PCR analysis of mRNA expression of early and (B) late myogenic markers. (C) Western blot analysis of myogenic markers Myogenin and Myosin heavy chain 2 in Septin9 deficient C2C12 cells compared to control. (D) Immunofluorescence stainings of Myosin heavy chain 2 (MF20) after 3 days of differentiation. (E) Myogenic differentiation index (MDI) and (F) myogenic fusion index (MFI) for cells in (D) respectively. N is the number of nuclei. Mononucleated cells are shown as reference. Data represent mean ± standard deviation (SD), ∗*p* < 0.05, ∗∗*p* < 0.01, ∗∗∗*p* < 0.001, ∗∗∗∗*p* < 0.0001 from two-sided unpaired t test. Scale bar 100 μm. See also Figure S9.

To understand whether an increased transcriptional signature associated with myogenic differentiation in Septin9-deficient cells translates into an accelerated or premature onset of myogenic differentiation, we conducted qRT-PCR analysis of early and late myogenic differentiation marker genes (Figures 3A and 3B). After a significant decrease in Septin9 expression (reduced to 30% in proliferating cells and 52% after 3 days of differentiation compared to control cells, Figure 2A), we observed a downregulation of Taz (Wwtr1) and an upregulation of Myogenin (Myog) expression in both proliferating and differentiating C2C12 cells upon induction of differentiation (Figure 3A). Subsequently, we noted a significant increase in the expression of terminal differentiation markers such as Follistatin (*Fst*) and two skeletal myosins, Myosin heavy chain 2 and 8 (*Myh2* and *Myh8*), which are typically not expressed in cycling myoblasts, but are induced during myogenic differentiation.^37^ This upregulation was observed after 72 h of differentiation in C2C12 cells upon Septin9 knockdown (Figure 3B). We further confirmed the elevated expression of Myogenin (after 24 h) and Myosin heavy chain 2 (Myh, after 72 h) at the protein level in response to Septin9 depletion (Figure 3C). Additionally, our analysis revealed that the loss of Septin9 led to an increase in the myogenic differentiation index (MDI) after 3 days of differentiation, as determined by quantifying the expression of Myosin heavy chain (Figures 3D and 3E). Furthermore, we found that myoblasts lacking Septin9 did not exhibit apparent fusion defects, as addressed by the myogenic fusion index (MFI, Figure 3F). In summary, the absence of Septin9 in myoblasts correlated with the premature activation of myogenic differentiation genes, concomitant with premature progreaation from proliferative to fusion-competent myoblasts.

### Septin9 reorganization during differentiation and fusion in C2C12 cells

**Figure 4:**
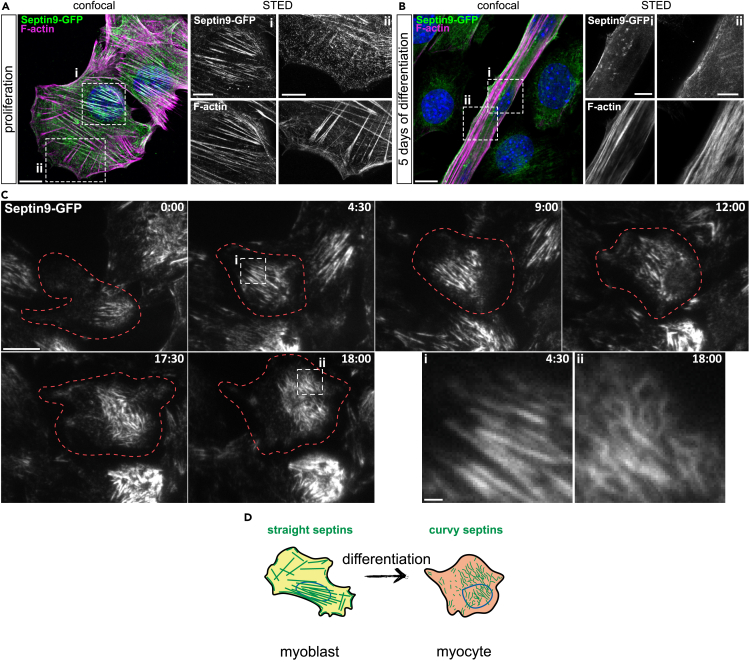
Septin9 reorganization during differentiation in Septin9-GFP C2C12 cells (A) Representative immunofluorescence micrographs showing septin filaments in proliferating Septin9-GFP C2C12 cells (A) and myotubes (B). Merge in confocal, inlays in STED. (C) Representative snapshots from a live cell TIRF experiment using differentiating Septin9-GFP C2C12 cells. Acquisition started 12 h after onset of myogenic differentiation, time in hours: minutes. Insets highlight changes in septin fiber morphology. (D) Schematic representation of a myoblast differentiating to a myocyte with according changes in septin morphology. Scale bar 10μm, insets 5 μm (A-B) and 1μm (C). inv-invading cells, rec-receiving cell. See also Figures S4–S8.

Myogenic differentiation requires a substantial reorganization of the myoblast cytoskeleton and the acquisition of a fusion-competent phenotype by myocytes.^37^^,^^38^^,^^39^^,^^40^^,^^41^ Therefore, we assessed the organization of Septin9-containing complexes during myogenic differentiation. To monitor the dynamic process of septin filament assembly, localization and rearrangement during myogenic differentiation, we generated a C-terminal meGFP-tagged Septin9 C2C12 cell line using CRISPR/Cas9 (Figure S4). In proliferating myoblasts, Septin9-GFP was observed in the basal plane to partially localize to perinuclear and ventral actin fibers, which were situated adjacent to focal adhesions (Figures 4A and S5A). Notably, Septin9-GFP was not found associated with dorsal actin fibers (Figure 4A, inset ii), consistent with previous observations in other cell lines.^32^ These findings align with previous research indicating that Septin9 directly cross-links actin filaments into bundles and facilitates the maturation of nascent focal adhesions.^42^ In contrast to flattened morphology of myoblasts, myotubes exhibited increased size and diameter, accompanied by distinct subcellular alterations in Septin9 distribution (Figures 4B and S6). Septin9 appeared to dissociate from actin filaments within myotubes, forming residual filament structures, such as short rods, spirals and rings. These structures were sporadically distributed in areas proximal to the plasma membrane along the myotube (Figures 4B and S6). Smaller myotubes lacking observable sarcomeric actin that resembled thick stress-like filaments exhibited a greater abundance of Septin9-structures in the basal plane, although, these structures appeared thinner, less organized and exhibited sparse colocalization with actin (Figure S6A). In mature myotubes with sarcomeric actin organization, patches of disorganized Septin9 remnants were evident in the basal plane (Figure S6B). The remaining space within the myotubes was predominantly occupied by actin-independent rings and membrane-associated patches of Septin9. This observed reorganization of Septin9 suggests its involvement in early myogenic differentiation.

To investigate whether the reorganization of Septin9 is associated with differentiation or fusion, we performed co-staining of Septin9 with the myogenic marker Myogenin after one day of differentiation (Figure S7). Myogenin-negative cells (Figure S7B, inset i) showed no discernible difference in Septin9 organization at day 1 compared to proliferating cells, with Septin9 decorating actin fibers. Similarly, the majority of Myogenin-positive cells (Figure S7B, inset ii-iii) exhibited actin-based Septin9 structures resembling those in proliferating cells, although they appeared more discontinuous, patchy and sporadically formed ring-like structures (Figure S7B, inset iii, arrowhead).

Furthermore, a subset of Myogenin-positive cells displayed pronounced reorganization of Septin9 into short, curvy rods that were no longer associated with actin filaments (Figure S7B, inset iv). These cells exhibited increased actin staining compared to undifferentiated or other Myogenin-positive cells, suggesting a higher degree of differentiation, as differentiated pre-fusion myoblasts were previously described to stain stronger for actin compared to undifferentiated cells.^41^ This subset of Myogenin-positive cells with the most pronounced Septin9 reorganization may represent a more advanced stage along the differentiation trajectory.

To better visualize the septin reorganization during early steps of myoblast differentiation we performed live cell experiments utilizing Septin9-GFP C2C12 cells. We employed total internal reflection (TIRF) microscopy to capture structural changes in septin organization near the plasmamemebrane as proliferating myoblasts transitioned into myocytes. Notably, we observed these events as early as 12 h into the differentiation process (Figures 4C and 4D; Video S1). Throughout myoblast differentiation, septins became more dynamic and curved, potentially disassociating from actin fibers (Figures 4C and 4D).

Video S1. TIRF imaging of differentiating Septin9-GFP C2C12 cells, related to Figure 4

**Figure 5:**
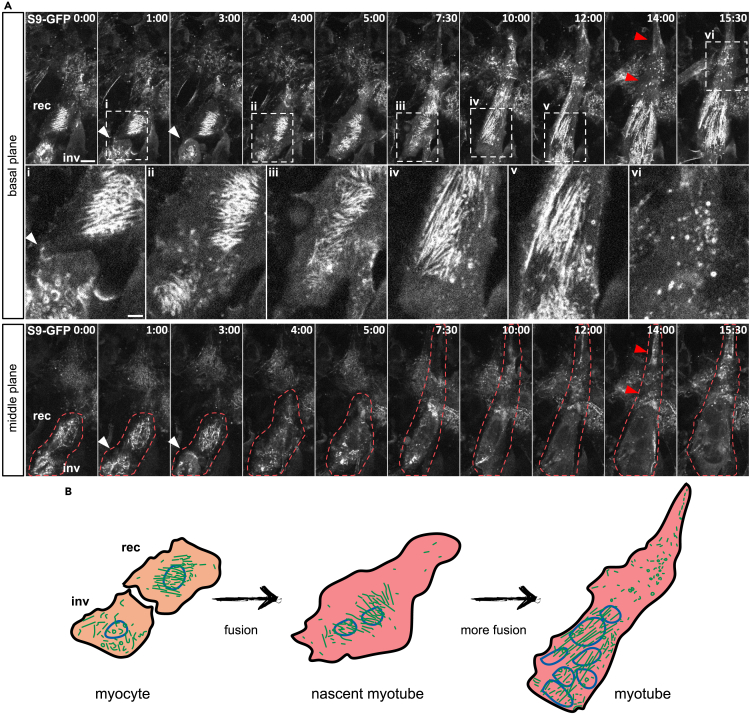
Septin9 reorganization during fusion in Septin9-GFP C2C12 cells (A) Representative snapshots from a live cell confocal experiment representing the basal and the middle plane of fusing Septin9-GFP C2C12 cells. Acquisition started 72 h after onset of myogenic differentiation, time in hours:minutes. Magnified insets show Septin9-GFP organization in the boxed area of the myotube. White arrow heads show Septin9-GFP at the leading edge of a lamellipodium. Red arrow heads show absence of Septin9-GFP in the distal part of a nascent myotube. (B) Schematic representation of a fusion event between to myocytes and the further maturation of the nascent myotube with according changes in septin morphology at several representative time points. Scale bar 10μm, insets 5 μm. inv-invading cells, rec-receiving cell.

During myocyte fusion, assessed via confocal microscopy after 3 days of differentiation (Figures 5A and 5B), we noted distinct septin organization in the invading versus the receiving myoblast at the basal plane. The receiving myocyte displayed curvy perinuclear septin filaments, while in the invading, fusion-competent myocyte, Septin9-GFP appeared reorganized into short rods and rings. These invading cells exhibited highly mobile and predominantly septin-free lamellipodia, known to play a role in the fusion process,^43^^,^^44^ with Septin9 observed at the leading edge (Figure 5A, white arrowheads at 1:00 and 3:00). After membrane fusion, perinuclear filaments mixed and reorganized, possibly to prepare for subsequent fusion event (Figure 5A, at 7:30; Videos S2 and S3). In the nascent myotube (Figure 5A, at 14:00 and 15:30), septin filaments extended across the nuclear region, while the remainder of the cell contained rings and other filament remnants (Figure 5A, red arrowheads at 14:00). The sarcoplasm, visible through the middle plane, appeared largely devoid of septins throughout the fusion process (Figure 5A, lower panels). From the live cell experiments, we conclude that actin-based septin filaments gradually disassemble from actin fibers as myoblasts undergo morphological changes toward myocytes. Myocytes adopt a migratory phenotype characterized by predominantly septin-free lamellipodia and short, curved septins. Newly formed nascent myotubes exhibit dynamic perinuclear fibers and only short filament remnants elsewhere in the cell body. Finally, the remaining septin structures gradually vanish from the basal plane of mature contractile myotubes.

Video S2. Basal plane confocal imaging of fusing Septin9-GFP C2C12 cells, related to Figure 5

Video S3. Middle plane confocal imaging of fusing Septin9-GFP C2C12 cells, related to Figure 5

## Discussion

The role of the septin cytoskeleton in the context of cell differentiation remains poorly understood. In our study, we have utilized myogenic differentiation as a paradigm and have demonstrated that septin structures are involved in modulating the pace of myogenic commitment. Furthermore, we have observed a profound reorganization of septin filaments during the transition from cycling myoblasts via fusion-competent myocytes to myotubes.

Our study highlights the expression of septin genes throughout the course of muscle regeneration and *in vitro* myogenic differentiation in C2C12 cells and primary myoblasts. Here, septin paralogues we found and their expression dynamics are generally in agreement with the ones reported by Gönczi et al.^11^ in juvenile and adult mouse muscles and C2C12 cells. Among these genes, we identified two core sets of myogenic septins consisting of the highest expressed paralogs, Septin2, 7, and 11 in quiescent MuSCs and Septin2, 7, 11 and 9 in all other sub-populations of MuSCs following injury. This suggests that quiescent and activated MuSCs may use a different set of septin protomers. Quiescent cells may rely on hexamers (Septin7-11-2), while cells that entered the cell cycle may require a more complex arrangement involving octamer-based heteromeric complexes (Septin9-7-11-2). Although, septins are believed to organize as paired octamer-based filaments at the interface between actin filament and plasma membrane,^32^ hexamer-based functions cannot be excluded and Septin9-positive and Septin11-positive filaments were reported to coexist in the cell during cell division.^45^ The protein expression and sub-cellular organization of different myogenic core and other septin paralogues in myogenic cells remain to be validated in future experiments.

Additionally, we reported on potentially regulatory septin paralogs, such as Septin4 and Septin6, which have been described as highly regulated differentially expressed genes in differentiating C2C12 cells.^46^ The exclusive upregulation of *Septin4* in the late commitment and fusion stages may signify a response to increased apoptotic resistance in mature myotubes,^47^^,^^48^^,^^49^ possibly involving the pro-apoptotic Septin4 splice variant Arts.^50^ The expression of *Septin6* in dividing MuSCs during muscle regeneration and its rapid downregulation during *in vitro* myogenic differentiation imply a particular function of this septin paralog during myoblast cell division. Other less abundant myoblast septins, including Septin5, 8 and 10 may play redundant roles, fine-tune septin filament composition, or as well engage in core filament-independent functions.

During early myogenic differentiation, proteins such as class II non-muscle myosins (NMIIA and NMIIB) and unconventional myosin VI (MVI, *Myo6*) have been reported to be expressed in myoblasts and to be subsequently downregulated.^37^ These proteins play crucial roles in regulating the progression of differentiation and fusion by modulating cytoskeletal organization.^41^^,^^51^^,^^52^ Specifically, depletion of MVI has been shown to induce alterations in cytoskeleton organization, cell adhesion, and promote fusion events in myoblasts derived from Snell’s waltzer mice, which exhibit a natural MVI knockout.^51^^,^^52^ It is noteworthy that core septins are known to interact with NMIIA, NMIIB and MVI in non-myogenic contexts.^21^^,^^53^^,^^54^ The shared expression pattern of septin paralogues and non-muscle myosins further support the notion that they likely participate in the regulation of myogenic differentiation.

Septin9 showed a distinct downregulation during regenerative myogenesis and *in vitro* myogenic differentiation and was used as a marker to track organizational changes during this dynamic process. Myoblasts express Septin9 which integrates into septin filaments, decorating contractile actin fibers within the basal plane of the cells. Upon the initiation of myogenic differentiation a notable reorganization and disassembly of Septin9 structures from actin filaments were observed in a subset of Myogenin-positive cells. Septins formed thin, curvy rods and rings, seemingly dissociated from actin. Upon fusion into myotubes, filament-like septin structures were observed in the basal perinuclear region, with a notable absence of other filamentous structures. Myotubes lacking pronounced sarcomeric actin organization predominantly exhibited actin-independent, curvy septin structures within the basal plane. However, mature myotubes displayed sporadic areas of septin remnants, primarily consisting of short, curvy rods and rings. As myotubes matured, basal septin structures increasingly diverged from observed in proliferating myoblasts. Various remnants of septin filaments were observed within 1 μm from the plasma membrane, while the remainder of the myotubal sarcoplasm contained septin rings and membrane-associated structures.

The alterations in septin filament organization can potentially be attributed to several factors, including the prevailence of splice variants, competition with actin-binding proteins, and changes in the affinity between septins and their interaction partners. The presence of differentially spliced isoforms of Septin9 within the octamer has been shown to influence subcellular localization and filament organization shifting from long to short filaments.^32^^,^^36^^,^^55^^,^^56^ Therefore, understanding the expression profile of Septin9 isoforms could provide insights into the isoform-specific regulation of filament integrity during myogenesis. Furthermore, Septin9 may face competition by myogenic actin-binding proteins such as skeletal myosins and actinins. Notably, throughout *in vitro* myogenesis Septin9, did not exhibit colocalization with α-actinin (Figure S5), which is involved into sarcomere formation and undergoes expression changes during myogenic differentiation.^57^

The initial reorganization of septin structures corresponds temporally with an observed increase in actin staining, coinciding with the emergence of aligned postmitotic pre-fusion myoblasts expressing skeletal myosin II.^37^^,^^41^ This actin remodeling during early myogenic differentiation facilitates crucial cellular changes, including myoblast migration, alignment and adhesion.^5^^,^^58^ The observed reorganization of Septin9 structures during early differentiation now underscores the involvement of septins in this process. While, septin assembly and cytoplasmic appearance can change during the migration of various cell types such as epithelia, fibroblasts, lymphocytes, neurons (reviewed in^17^) and myoblasts,^12^ the disassembly of Septin9 from actin observed in our study at the onset of differentiation does not parallel migration processes. The reduced association of Septin9 with actin during differentiation may be explained by shifts in septin affinity toward microtubules or the plasma membrane.^36^^,^^59^^,^^60^^,^^61^^,^^62^ While septins only occasionally colocalize with microtubules in myoblasts and myotubes (Figure S8), they may translocate to the plasma membrane, moving away from the sarcomeric machinery and toward the increasingly dynamic cell surface of committed, fusion-competent progenitors.^63^^,^^64^

The distinct distribution pattern of septins within myotubes poses several intriguing questions. It will be important to elucidate temporal dynamics underlying the formation of septin rings observed within the sarcoplasm, discerning whether they emerge at the onset of differentiation or persist over subsequent days. Additionally, it will be crucial to understand the relationship between Septin9 remnants in the subsarcolemmal space and regions of myotube-substratum adhesion, as well as to determine if their association with the non-sarcomeric muscle cytoskeleton is essential for internal myotube architecture.^65^^,^^66^^,^^67^ Previous research by Gönczi et al. highlighted the co-localization of Septin7 with mature t-tubules and suggest a role in adult muscle architechture.^11^ Whether Septin9 is incorporated into these mature structures, and their composition, warrant investigation. Moreover, exploring the interaction between sarcomeric septin structures and the non-sarcomeric cytokskeleton, as well as the potential role of septins in myofibrillogenesis pose further questions.

Our study reveals a temporary increase in myogenicity following Septin9 depletion during early myogenic differentiation in both C2C12 cells and primary myoblasts. This contrasts with prior studies demonstrating deleterious effects of Septin7 depletion on myogenic differentiation in C2C12 cells.^11^ The discrepancy in outcomes may stem from impaired progression through cell division upon depletion of either Septin9 or Septin7. Septin2, 7, 9 and 11 are integral to cell division,^68^^,^^69^^,^^70^ with few exceptions such as certain hematopoietic cells that employ septin-independent mechanisms for cytokinesis (reviewed in^71^). Notably, Septin7 depletion abolishes the formation of any septin structures, leading to cytokinetic failure and a spectrum of cellular defects, as evidenced by previous research.^11^ This disruption in cell pool expansion and viability likely contributes to the observed effect on myogenic differentiation.^11^ Conversely, Septin9 has been shown to be essential for the late stage of cytokinesis in HeLa and MDCK cells.^68^^,^^69^^,^^70^ Surprisingly, we did not observe cytokinetic defects upon Septin9-depletion in C2C12 cells and primary myoblasts, consistent with similar finding in murine fibroblasts, where Septin9 depletion resulted in a minor increase in polynucleation but had no impact on cell proliferation.^31^ Although robust depletion of Septin9 results in a significant reduction in Septin9 expression and the absence of Septin7 and Septin2-based structures (Figure S9), cells may potentially overcome cytokinetic defects by utilizing the remaining levels of Septin9 or by transitioning to a hexamer-based protofilament.

Furthermore, the discrepancy in observed outcomes may potentially be attributed to alterations in migratory phenotypes resulting from Septin9 or Septin7 depletion. Inhibition of myoblast migration has been demonstrated to enhance myoblast fusion and, consequently, could potentially influence differentiation.^72^ As demonstrated by Gönczi et al., Septin7 depletion enhances myoblast migration, providing support for the decline in myogenic differentiation in Septin7-deficient myoblasts.^11^ Septin9 has previously been implicated in enhancing migration in renal and mammary epithelial cells.^42^^,^^73^^,^^74^ Additionally, Farrugia et al. reported paralogue-specific differences in melanoma cells undergoing ameboid migration, where Septin9, but not Septin7 influenced the migration phenotype.^75^ However, we have not investigated the migration of Septin9-deficient myoblasts, leaving this aspect to be elucidated in future studies.

Due to their ability to break symmetry in cells,^76^ septins are believed to affect the balance between self-renewal and differentiation or the mode of stem cell division (symmetric or asymmetric).^77^ In hematopoietic stem cells, Septin7 plays a critical role by compartimenatlizing cytoplasmic cell polarity markers like Cdc42 and Borg4, thereby regulating the potential for hematopoietic differentiation.^78^ Moreover, Septin7 is involved in the maintainance of the motor protein Kif20a at the intracellular bridge of neuronal progenitor cells (NPCs). This involvement suggests a role in controlling the inheritance of neuronal cell fate determinants by daughter cells. The depletion of Septin7 leads to premature neuronal differentiation, disrupting the balanced regulation of asymmetric cell division.^79^^,^^80^ These findings raise the possibility that transit-amplifying cells, such as myoblasts, may also possess a limited capacity of asymmetric division, or a similar mechanism of cell fate decision tied into the balance between proliferation and differentiation. Recent research has revealed that in myotubes derived from C2C12, Septin7 interacts with Numb, another cell fate determinant.^14^ Therefore, it is possible that septin complexes may compartmentalize cell fate determinants in myogenic cells through a mechanism resembling neuronal or hematopoietic progenitors. It is conceivable that the role of septins in cell fate determination is conserved across different cell types.

In summary, we show that the septin cytoskeleton is an additional layer of regulation controlling the progression of myogenic differentiation. We propose that the controlled reorganization of septin structures is instrumental in transitioning from an undifferentiated, proliferative myoblast state to a fusion-competent terminally differentiated state.

### Limitations of the study

A potential limitation of the study lies in the challenge of untangling the mutually instructing nature of septin-actin interactions. Our *in vitro* data show changes in expression of septin paralogs, which are preceded by morphological changes in the organization of septin filaments. This process may potentially coincide with actin reorganization. Since septins are known to regulate actin stability, bundling and organization, the observed effect of septin depletion might exert its myogenic regulation through impaired actin function.^56^^,^^81^

## STAR★Methods

### Key resources table

**Table:** 

REAGENT or RESOURCE	SOURCE	IDENTIFIER
**Antibodies**		

Mouse monoclonal anti-MF20, dilution IF 1:100, WB 1:500	DHSB	Cat# MF 20; RRID:AB_2147781
Mouse monoclonal anti-Myog, dilution WB 1:100	Santa Cruz	Cat# sc-12732; RRID:AB_627980
Mouse monoclonal anti-Myog, dilution IF 1:100	DHSB	Cat# F5D; RRID:AB_2146602
Mouse monoclonal anti-Vinculin, dilution WB 1:1000	Sigma-Aldrich	Cat# V9131; RRID:AB_477629
Mouse monoclonal anti-Actinin-2, dilution IF 1:500	Sigma-Aldrich	Cat# A7811; RRID:AB_476766
Mouse monoclonal anti-Actin beta, dilution WB 1:5000	Sigma-Aldrich	Cat# A5441; RRID:AB_476744
Mouse monoclonal anti-alpha Tubulin, dilution IF 1:500	Cell Signaling	Cat# 3873; RRID:AB_1904178
Rabbit monoclonal anti-Gapdh, dilution WB 1:2000	Cell Signaling	Cat# 2118, RRID:AB_561053
Rabbit polyclonal anti-Septin9, dilution IF 1:500, WB 1:2500	Homemade; Kind gift from Prof. Dr. M. Krauss^82^	N/A
Rabbit polyclonal anti-Septin2, dilution IF 1:500	Sigma-Aldrich	Cat# HPA018481, RRID:AB_1856684
Rabbit polyclonal anti-Septin7, dilution IF 1:500	IBL America; Kind gift from Prof. Dr. Helge Ewers	Cat# IMB-18991, RRID:AB_10700085
Rabbit polyclonal anti-GFP, dilution IF 1:1000	Abcam	Cat# ab6556, RRID:AB_305564
Mouse monoclonal anti-GFP, dilution IF 1:100, WB 1:100	Santa Cruz	Cat# sc-9996, RRID:AB_627695
Polyclonal goat anti-mouse IgG (H+ L), dilution WB 1:5000	Dianova	Cat# 115-035-068, RRID:AB_2338505
Polyclonal goat anti-rabbit IgG (H+ L), dilution WB 1:5000	Dianova	Cat# 111-035-144, RRID:AB_2307391
Polyclonal goat anti-rabbit IgG (H+ L) AF488, dilution IF 1:400	Thermo Fisher Scientific	Cat# A-11034, RRID:AB_2576217
Polyclonal goat anti-rabbit IgG (H+ L) AF594, dilution IF 1:400	Thermo Fisher Scientific	Cat# A-11012, RRID:AB_2534079
Polyclonal goat anti-mouse IgG (H+ L) AF488, dilution IF 1:400	Thermo Fisher Scientific	Cat# A-11001, RRID:AB_2534069
Polyclonal goat anti-mouse IgG (H+ L) AF594, dilution IF 1:400	Thermo Fisher Scientific	Cat# A-11005, RRID:AB_2534073

**Bacterial and virus strains**		

DH5α Chemically Competent E. coli	Our lab	N/A

**Chemicals, peptides, and recombinant proteins**		

Phalloidin CruzFluor™ 594 Conjugate, dilution IF 1:600	Santa Cruz	Cat# sc-363795
Phalloidin CruzFluor™ 647 Conjugate, dilution IF 1:600	Santa Cruz	Cat# sc-363797
Fetal Bovine Serum (FBS)	PAN Biotech	Cat# P30-1302
Horse serum (HS)	Gibco, Thermo Fisher Scientific	Cat# 16050122
Recombinant Human FGF-basic (154 a.a.)	PeproTech	Cat# 100-18B
Matrigel® Basement Membrane Matrix, LDEV-free	Corning	Cat# 354234
Chick Embryo Extract	LSP	Cat# MD-004D-UK
Lipofectamine RNAiMAX	Thermo Fisher Scientific	Cat# 13778150
Luna® Universal qPCR Master Mix	New England BioLabs	Cat# M3003L
M-MuLV reverse transcriptase enzyme	New England BioLabs	Cat# M0253S
Collagen I, rat tail	Gibco, Thermo Fisher Scientific	Cat# A1048301

**Critical commercial assays**		

WesternBright Quantum kit	Advansta	Cat# K-12042-D10
NucleoSpin RNA isolation kit	Macherey-Nagel	Cat# 740955.250
NEBuilder® HiFi DNA Assembly Cloning Kit	New England BioLabs	Cat# E5520S

**Deposited data**		

Bulk mRNASeq upon Septin9 depletion in C2C12 and primary myoblasts	This manuscript	GSE272316

**Experimental models: Cell lines**		

C2C12 cells	ATCC	CRL-1772
Septin9-eGFP C2C12 cells	This manuscript	Available upon request
Primary mouse myoblasts, hindlimbs, 5weeks old	This manuscript	N/A

**Oligonucleotides**		

ON-TARGETplus Non-targeting Control siRNA #1	Dharmacon	Cat# D-001810-01-50
ON-TARGETplus Mouse Septin9 siRNA, CCAACGGCATTGACGTGTA	Dharmacon	Cat# J-048947-11-0050
Primers Real-time PCR, Table S1	ordered at Thermo Fisher Scientific	N/A

**Recombinant DNA**		

pSpCas9(BB)-2A-GFP (PX458)	Addgene	Cat# 48138; RRID:Addgene_48138

**Software and algorithms**		

Prism (v9.3)	GraphPad Software	https://www.graphpad.com/scientific-software/prism/
NEBuilder Assembly Tool	New England BioLabs	https://nebuilder.neb.com/#!/
R (v4.0.5)	R Core Team	https://www.r-project.org/
Fiji	Schindelin et al.^83^	https://imagej.net/

**Other**		

μ-Slide 8 Well	Ibidi	Cat# 80826

### Resource availability

#### Lead contact

Further information and requests for resources and reagents should be directed to and will be fulfilled by the Lead Contact, Petra Knaus (petra.knaus@fu-berlin.de).

#### Materials availability

This study did not generate new unique reagents. Septin9-GFP C2C12 cell line used in this study is available upon reasonable request.

#### Data and code availability

• Data reported in this paper will be shared by the lead contact upon reasonable request.
• Bulk mRNA-seq data have been deposited at GEO and are publicly available as of the date of publication. Accession number is listed in the key resources table.
• This paper does not report original code.
• Any additional information required to reanalyze the data reported in this paper is available from the lead contact upon reasonable request.

### Experimental model and study participant details

#### Mouse cell lines

C2C12 were obtained from American Type Culture Collection (ATCC) and not used beyond passage 25. C2C12 and Septin9-GFP C2C12 cell lines (generation is described below) were cultured in Dulbecco’s Modified Eagle’s Medium (DMEM) containing 4.5 g/L D-glucose and phenolred (PAN Biotech), supplemented with 10% FBS, 2 mM L-glutamine and penicillin (100 units/mL) / streptomycin (100 μg/mL) (C2C12 full medium) in a humidified atmosphere at 37°C and 10% CO_2_ (v/v). To induce differentiation in C2C12 cells, proliferation medium was removed and replaced with differentiation medium: DMEM containing 4.5 g/L D-glucose and phenolred, 2% HS (Gibco), 2 mM L-glutamine and penicillin (100 units/mL) / streptomycin (100 μg/mL). Primary myoblasts were cultured in DMEM containing 4.5 g/L D-glucose, phenolred and stable L-glutamine, supplemented with20% FCS, 10% HS, 2.5 ng/ml recombinant human bFGF (PeproTech), 0.5% chicken embryo extract (LSP) (myoblast proliferation medium) in a humidified atmosphere at 37°C and 5% CO_2_ (v/v). To induce differentiation in primary myoblasts, proliferation medium was removed and replaced with differentiation medium: high glucose DMEM, 5% HS and penicillin (100 units/mL) / streptomycin (100 μg/mL).

### Method details

#### Primary myoblast isolation

Mice were kept in accordance with European Union and German legislation under the license number ZH120. Skeletal muscle progenitors were isolated from hindlimbs of 5 weeks old mice as described in.^84^ In brief, muscles were minced and digested with 2.5 mg/ml collagenase A (Roche) for 1 h at 37°C. Cells were centrifuged at 300 g for 5 min and supernatant was removed. Cell pellet was resuspended in myoblast proliferation medium containing 20% FCS, 10% HS, 2.5 ng/ml recombinant human FGFb (Peprotech), 0.5% chicken embryo extract (LSP). The pellet was placed on Matrigel (Corning) coated plates (working concentration of 0.9 mg/ml) where myoblast migration from the minced myofibers was observed on day 3 of culture. At that point, cells were trypsinized and plated for 1 h on type I rat tail collagen (working concentration 0.1 mg/ml) (Corning) coated plates for removal of fibroblastic and non-myogenic cells. Next, medium containing non-attached cells was collected and placed on Matrigel coated plates for expansion of the myoblast culture.

#### Transient transfection with siRNA

Septin9 was silenced in C2C12 using one round of 48h knock-down with 50 nM siRNA, purchased from Dharmacon. Cells were transfected with either scrambled (non-targeting #1) siRNA or Septin9 siRNA (J-048947-11-0050 ONTARGETplus) with Lipofectamine-iMAX (ThermoFisher Scientific) according to the manufacturer’s instructions. In brief, 150.000 cells / 6cm-dish were seeded in 2 ml antibiotic-free medium. On the following day, siRNA – Lipofactamine mix was prepared in Opti-MEM™ - Reduced Serum Medium (ThermoFisher Scientific) and incubated for 20 min. Cells were washed with PBS once and 1.7 ml Opti-MEM™ was added. Next, 300 μl transfection mix was added dropwise to the cells (calculated for total 4 ml transfection medium), effectively increasing the siRNA concentration to 100 nM for the first 5 hours. Subsequently, after 5h 2 ml 20% FCS antibiotic-free medium was added. 24 h later the cells were trypsinized, re-plated for the experiment and the medium was replaced with full proliferation medium. All experiments were performed 48 h after siRNA transfection. Primary myoblasts were handled at recommended densities^84^ and transfected in 3.7 ml full myoblast proliferation medium and 300 μl Opti-MEM/siRNA mix. 24 h later cells were re-plated for the experiment and the medium was exchanged for fresh proliferation medium.

#### SDS-PAGE & western-blotting

For sodium dodecyl sulfate polyacrylamide gel-electrophoresis (SDS-PAGE), treated cells were trypsinized, washed with PBS, and lysed for 15 minutes on ice in RIPA light lysis buffer (25 mM Tris, 150 mM NaCl, 0.1 % SDS, 0.5 % IGEPAL (v/v)), supplemented with protease inhibitor cocktail (Roche) and 1mM PMSF. The lysates were then passed through a 20-gauge syringe and cleared by centrifugation at 11000g for 15 minutes at 4°C. Protein concentration was measured using the BCA test (Thermo Fisher Scientific). Lysates were denatured in Laemmli buffer for 5 minutes at 95°C. 10% polyacrylamide gels were cast in advance and stored at 4°C until use. For each sample, 10-30 μg of protein were separated by molecular weight. Proteins were transferred onto nitrocellulose membranes via Western blot. Membranes were blocked for 1 hour in 0.1% TBS-T containing 5% w/v skim milk, washed three times in 0.1% TBS-T and incubated with primary antibodies overnight at 4°C. Primary antibodies were applied in 3% w/v bovine serum albumin (BSA)/fraction V in TBST. For HRP-based detection, goat-α-mouse or goat-α-rabbit IgG HRP conjugates (± 0.8 mg/ml) were used in 3% TBST. Chemiluminescent reactions were processed using WesternBright Quantum HRP substrate (Advansta) and documented on a FUSION FX7 digital imaging system.

#### Generation of a eGFP-Septin9 knock-in C2C12 cell line

Endogenous tagging of the Septin9 C-terminus with meGFP was achieved via CRISPR/Cas9.^85^ In brief, the primers 5′-AAACCTGGACCCCACCCCCAGATC-3` and 5′- CACCGATCTGGGGGTGGGGTCCAG-3′ were annealed and cloned in the px458- pSpCas9(BB)-2A-GFP (Addgene, #48138) using the BpiI restriction site to generate a guide RNA. In a second (“donor”) vector the expression cassette was exchanged with the coding sequence (CDS) of meGFP inserted between two homology regions (HR) consisting of original genomic sequences ∼ 1000 bp upstream (5′HA) and ∼1000 bp downstream (3′HA) of the Septin9 stop codon, separated by a Gly-Ser-Gly-Ser-Gly linker (L). Stop codon of the Septin9 was removed on the donor vector. Design and cloning of the donor vector was performed with the NEBuilder assembly tool and NEBuilder HiFi DNA assembly cloning kit, respectively, according to the manufacturer instructions. C2C12 cells were transfected with px458-pSpCas9(BB)-2A-GFP and donor vector. 72h later GFP-expressing C2C12 cells were sorted into 96-well plates at the density of 1 cell per well, using a fluorescence-activated single cell sorter (BD FACSAriaII SORP (BD Biosciences) at MPI-MG Berlin). Growing colonies were expanded and tested for the expression of Septin9-eGFP by immunofluorescence and Western blotting using anti-Septin9 and anti-GFP antibodies. The expression of Septin9-eGFP in selected clones was further validated by siRNA-mediated depletion of Septin9 and immunocytochemistry.

#### Immunocytochemistry

C2C12 cells seeded on 8 μ or 18 μ Ibidi chambers or 12 mm cover slips were fixed with 4% PFA in PBS for 15 minutes at RT and washed two times with PBS. Permeabilization with 0.5% Triton X-100 in PBS was done for 20 minutes, subsequently cells were blocked with blocking solution (5% normal goat serum, 3% BSA in PBS) for 1 hour. Primary antibodies were incubated in the blocking solution for 1 h at RT or overnight at 4°C, excess of antibody was removed with three washing steps of 5 minutes with 3% BSA in PBS. Incubation with secondary antibodies was carried out in blocking solution for 1 h at RT with three subsequent washing steps. Finally, cells were incubated for 5 minutes with 1 μg/mL of DAPI in PBS and washed with water. 18 μ Ibidi chambers were left in PBS, coverslips were mounted on microscope glass slides with FluoromountG (Invitrogen) or Prolonged Gold (for STED microscopy).

#### Confocal & STED microscopy

Confocal and STED data of fixed C2C12 cells were acquired with the Expert Line STED Microscope from Abberior. Confocal images of Septin9-GFP C2C12 cells were acquired using 485 nm (20% laser power) and 640 nm excitation (20% laser power). STED images were acquired using 561 nm excitation for ATTO 594 (20% laser power) and 640 nm excitation for a 775 nm STED laser at 10% laser power was used to deplete both dyes.

#### Live cell microscopy

Live cell imaging was performed in differentiation medium without phenolred at 37°C and 5% CO2. Confocal live cell imaging of Septin9-eGFP cells was performed on a spinning disk Nikon Eclipse Ti microscope (Yokogawa CSU-X1 and EMCCD Camera), operated by NIS-Elements software, with a 40x air objective (0.75 NA). Imaging was initiated 72 h after medium change, and was carried overnight, with a frame rate of 30 minutes. Pictures were acquired within two z-planes that were set at the beginning by focusing on septin fibers and 0.5 μm above, and kept by an autofocus system. TIRF live cell imaging was conducted with a Nikon Eclipse Ti microscope (illumination: TIRF laser 488, prime95B sCMOS camera) operated by Micromanager, using a 60x oil objective (1.49 NA). Imaging was initiated after medium change, and was carried overnight, with a frame rate of 30 minutes.

#### Image analysis & semi-automated quantification with Fiji

For Figure 3 at least 3 independent experiments with 3 technical replicates from the 8 μ Ibidi chamber slide were performed. Tile scans of 6.4 (4x4) or 3.6 (3x3) μm^2^ were produced by stitching together images automatically acquired around a chosen point with 20x objective. Four to five tile scans were acquired per condition. The progress of myogenesis was quantified by counting nuclei within and outside of myotubes using a custom-written Fiji macro. Both myotubes and nuclei were fluorescently labeled (MF20 and DAPI), and a Gaussian blur with a sigma radius of 1 pixel was applied to smooth the color channels. The Otsu-based thresholding method was employed to separate the nuclei from the background, and a watershed algorithm was utilized to segment touching nuclei. Morphological operations including despeckle, 4x dilate, close, fill holes, and 3x erode were performed on the stained myotubes to address any irregularities. Additionally, touching myotubes were segmented using a watershed algorithm. The processed myotube channel was used to define regions of interest (ROIs), and the number of nuclei was counted for each myotube within these defined regions. To quantify the total number of nuclei, both within and outside of the myotubes, Fiji’s Particle Analyzer was employed.^83^ To ensure the accuracy of the analysis, a composite image combining the fluorescence channels of the myotubes and nuclei was generated. The outlines of the analyzed myotubes and nuclei were overlaid on this image, allowing for manual validation of the analysis quality.

#### Quantitative real-time PCR

Myogenic differentiation experiments with C2C12 and primary myoblasts were initiated by a medium change for serum-reduced differentiation medium after cells reached 90% confluency. For sample collection, cells were rinsed twice with PBS and lysed in 350 μl RA1 buffer (RNA extraction kit), supplemented with 1% β-mercaptoethanol, stored at -80°C until further processing. Cellular RNA was isolated using the NucleoSpin RNA isolation kit (Macherey-Nagel, Düren, Germany) according to the manufacturer’s instructions. 1 μg total RNA was reversely transcribed by incubation with random primers (100 pmol μL^–1^, Invitrogen, Carlsbad, USA) and M-MuLV reverse transcriptase enzyme (200,000 U mL^–1^, New England Biolabs, Ispwich, USA) was added per sample. RT-PCR was performed using a StepOnePlus Real-Time PCR System (Thermo Fisher Scientific) with specific primers (Table S1). Reactions were performed in triplicates in MicroAmp Optical 96-well reaction plates (Thermo Fisher Scientific) using Luna PCR Master Mix (New England Biolabs). Fold induction was calculated by comparing relative gene expression to the housekeeping gene 18S RNA using the ΔΔCT method.^86^

#### Single-nucleus RNA-sequencing data analysis

Single-cell sequencing data was retrieved from the Gene Expression Omnibus (GSE138826) as a pre-annotated data frame containing RNA counts values. Data was first log-normalized using a scale factor of 10^4^. The top 2000 variable genes were identified by the *FindVariableGenes()* function using the default settings. Centering and scaling of the data using a linear regression model was then performed on the previously identified 2000 variable features. For clustering, a shared nearest neighbor graph was constructed using the first 25 principal components (PCs). Louvain clustering was performed with the clustering resolution set to 0.5. Overall cluster annotation was available in the data frame retrieved from GEO.

#### Subclustering of single-cell sequencing data

Cells attributed to “MuSC” were selected and the resulting subgroup of cells was scaled and a shared nearest neighbor graph was constructed using the first 10 principal components (PCs). Louvain clustering was performed with the clustering resolution set to 0.4. Subclusters were annotated based on expressed marker genes. The top three marker genes upon which cell identities were assigned are as follows: quiescent cells are marked by Pax7, Sdc4, and Spry1; activated cells by Pax7, Islr, and Itm2a; dividing cells by Top2a, Cdk1, and Pax7; and differentiated cells by Myog, Ttn, and Myl4. Notably, 2 clusters did not express essential myogenic markers and were therefore excluded for further analysis. Similar to the original publication, we identified a cluster of immuno-myoblasts (expression of C1qa) that we did not display for enhanced comprehensiveness of our plots.

#### RNA-seq library preparation and sequencing

40.000 cells/cm^2^ cells were seeded in a 12 well plate 24 hours after Septin9 knockdown in full myoblast proliferation medium from two independent experiments. Cells were expanded for 24 hours (total 48 hours after knockdown). Primary myoblasts were lysed, and C2C12 cells differentiated for 12 hours and then lysed. RNA was isolated according to the manufacturer instructions (NucleoSpin RNA isolation kit (Macherey-Nagel)). 500ng RNA was sent for sequencing to Genewiz, Leipzig, Germany.

#### RNA-seq data analysis

Paired-end, 300-bp reads from Illumina sequencing were mapped to the GRCm38.p6 reference genome with STAR (v2.7.2b)^87^ and default settings using the Galaxy platform.^88^ Raw count matrices were built using featureCounts (v2.0.1) with a GTF annotation file for GRCm38 from Gencode (vM15).^89^ Subsequently, batch effects in the resulting raw count data were modeled with ComBat-Seq^90^ and gene expression was quantified using DESeq2^91^ on wild-type versus knock-down samples with batch effects removed on the ComBat-Seq adjusted count matrices. Gene ontology enrichment analysis was performed with Enrichr.^92^ Subsequent data visualization was performed in R (v4.1.1) using the pheatmap,^93^ ggplot2,^94^ ggrepel,^95^ and dplyr^96^ packages.

### Quantification and statistical analysis

All data are derived from at least three independent experiments (except RNA-seq) and are represented as means± standard deviation (SD). Statistical tests were performed using GraphPad Prism (v9.3) software. All statistical tests are listed in the figure legends. Datasets were tested for normality with the Shapiro-Wilk test. Two tailed student’s t-test was used to compare between two conditions. Whenever comparing more than two conditions, the one-way ANOVA and Dunnett post hoc test were used to check for statistical significance under the normality assumption. The level of significance is indicated in the figures by asterisks (∗P < 0.05; ∗∗P < 0.01; ∗∗∗P < 0.001; ∗∗∗∗P < 0.0001).

## Supplemental information

Document S1. Figures S1–S9 and Table S1

Table S2. RNASeq normalized counts C2C12, related to Figure 2

Table S3. RNASeq normalized counts primary myoblasts, related to Figure 2

Table S4. RNASeq differential expression values C2C12, related to Figure 2

Table S5. RNASeq differential expression values primary myoblasts, related to Figure 2
