# Supplementary material for: Dynamic remodeling of septin structures fine-tunes myogenic differentiation
Source: iScience. 2024 Jul 31;27(9):110630. doi: 10.1016/j.isci.2024.110630 (PMC11380178; doi:10.1016/j.isci.2024.110630)
Supplement: Document S1. Figures S1–S9 and Table S1 [file mmc1.pdf]

iScience, Volume *27*

Supplemental information

Dynamic remodeling of septin structures

fine-tunes myogenic differentiation

Vladimir Ugorets, Paul-Lennard Mendez, Dmitrii Zagrebin, Giulia Russo, Yannic Kerkhoff, Georgios Kotsaris, Jerome Jatzlau, Sigmar Stricker, and Petra Knaus

Supplementary Figures

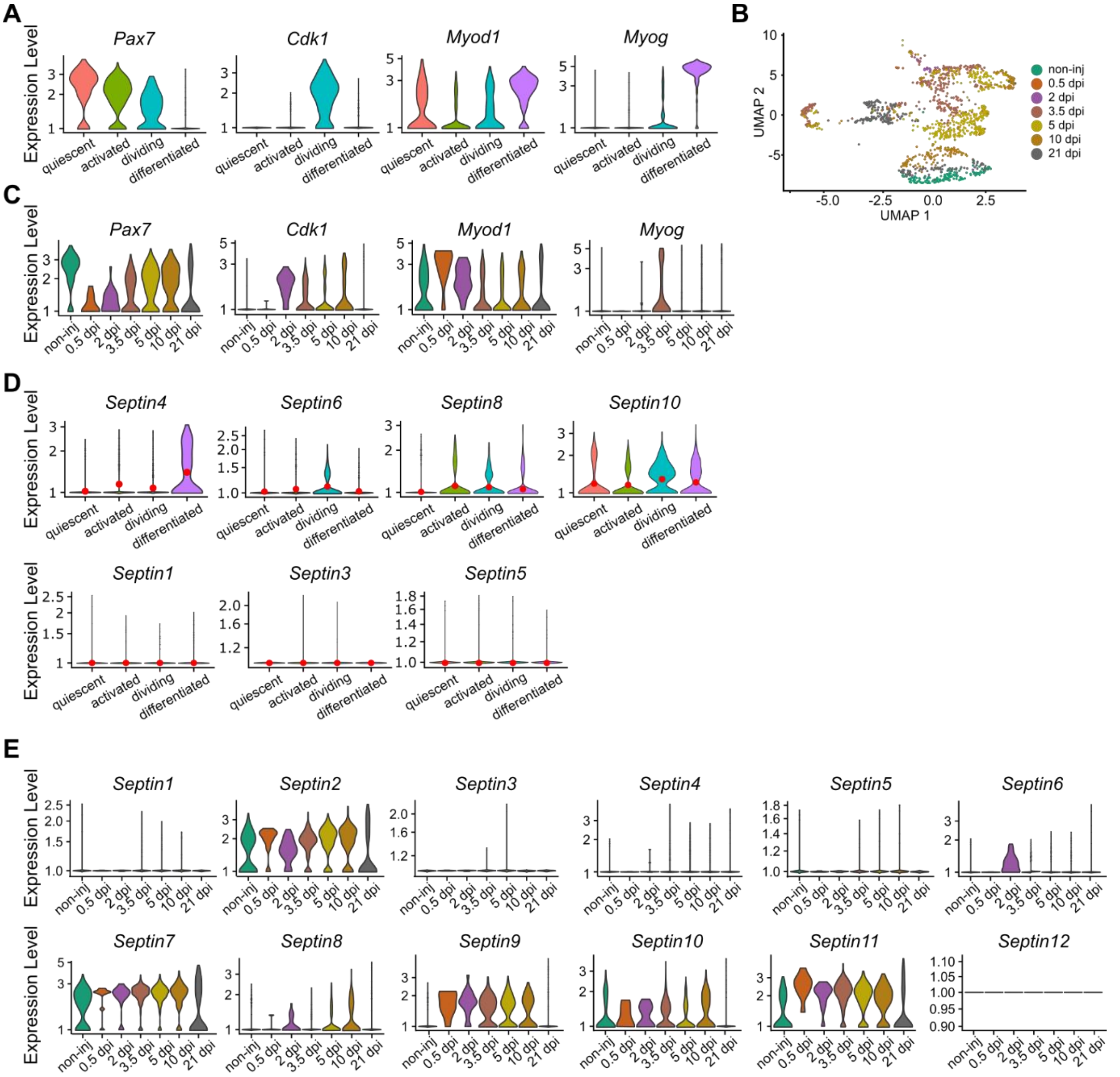

Figure S1 Transcriptional landscape of septins in the population of MuSCs during muscle regeneration, Related to Figure 1.

Single cell RNA sequencing data of non-injured tibialis anterior muscle and additional six time points following cardiotoxin injury (CTX)[1]. **A** Violin plots representing subcluster-specific gene expression colored by cluster identity. **B** UMAP visualization of the sub-cluster MuSC in regenerating musculature colored by time points within the sub-cluster. **C** Violin plots representing subcluster-specific gene expression colored by time points. D Violin plots showing time point specific expression of septins during muscle regeneration. **D** Violin plots representing gene expression profiles of *Septin1, 3, 4, 5, 6, 8* and *10* in MuSC subclusters. **E** Violin plots representing subcluster-specific gene expression of all septins colored by experimental time points.

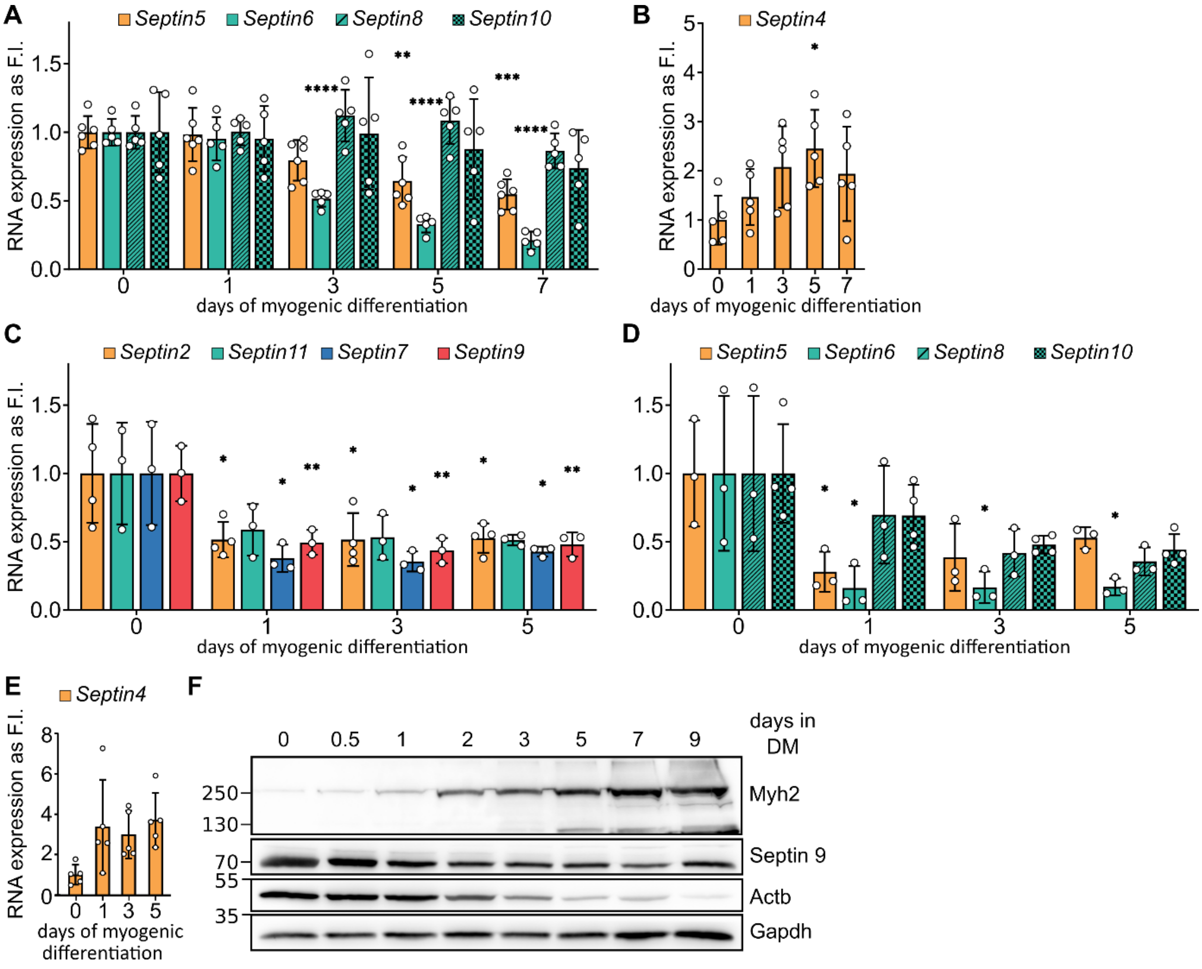

Figure S2 Expression of septins during myogenic differentiation in C2C12 cells and primary myoblasts, Related to Figure 1.

A-B mRNA expression levels of *Septin5*, *6*, *8*, *10* and *4* during C2C12 differentiation. **C-E** mRNA expression levels of core myogenic septins (C) and other expressed septin subunits (D-E) during 5 days of myogenic differentiation in primary myoblasts. **F** Representative western blot of Septin9 during 9 days of myogenic differentiation in primary myoblasts. Data represent mean ± standard deviation (SD), *p<0.05, **p<0.01, ***p<0.001, ****p<0.0001 from one way ANOVA followed by Dunnett's multiple comparison test.

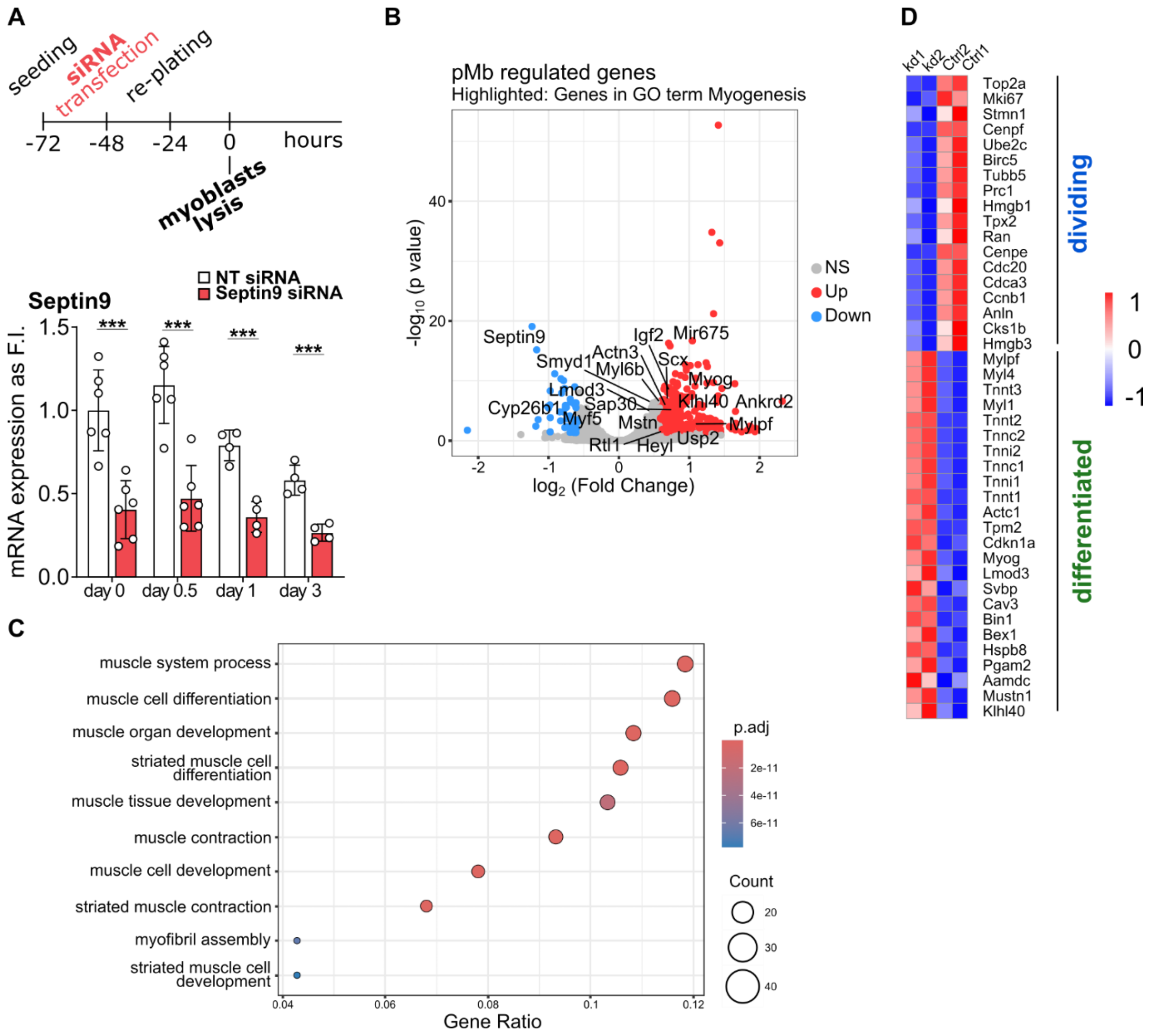

Figure S3 Septin9 depletion induces transition from cycling towards committed cells in primary myoblasts, Related to Figure 2.

A Schematic representation of the experimental setup. Total RNA was isolated from proliferating myoblasts 48 hours after Septin9 knockdown. Septin9 knockdown efficiency validation over 3 days of *in vitro* myogenic differentiation via qRT-PCR in primary myoblasts. **B** Volcano plot of DE genes (adjusted p value < 0.05; -0.585 ≤ log2FC ≤ 0.585) of primary myoblasts transfected with either non-targeting siRNA (control) or Septin9 siRNA after in growth medium, genes corresponding to Gene Ontology term "Myogenesis" are highlighted. **C** GO enrichment analysis of genes upregulated in Septin9-deficient primary myoblasts. **D** Heat map depicting marker genes for dividing and differentiated MuSCs defined by scRNAseq data depicted in Figure 1. Genes are filtered for significant regulation in primary myoblasts (adjusted p value <0.05). Data represent mean ± standard deviation (SD), *p<0.05, **p<0.01, ***p<0.001, ****p<0.0001 from two-sided unpaired t test.

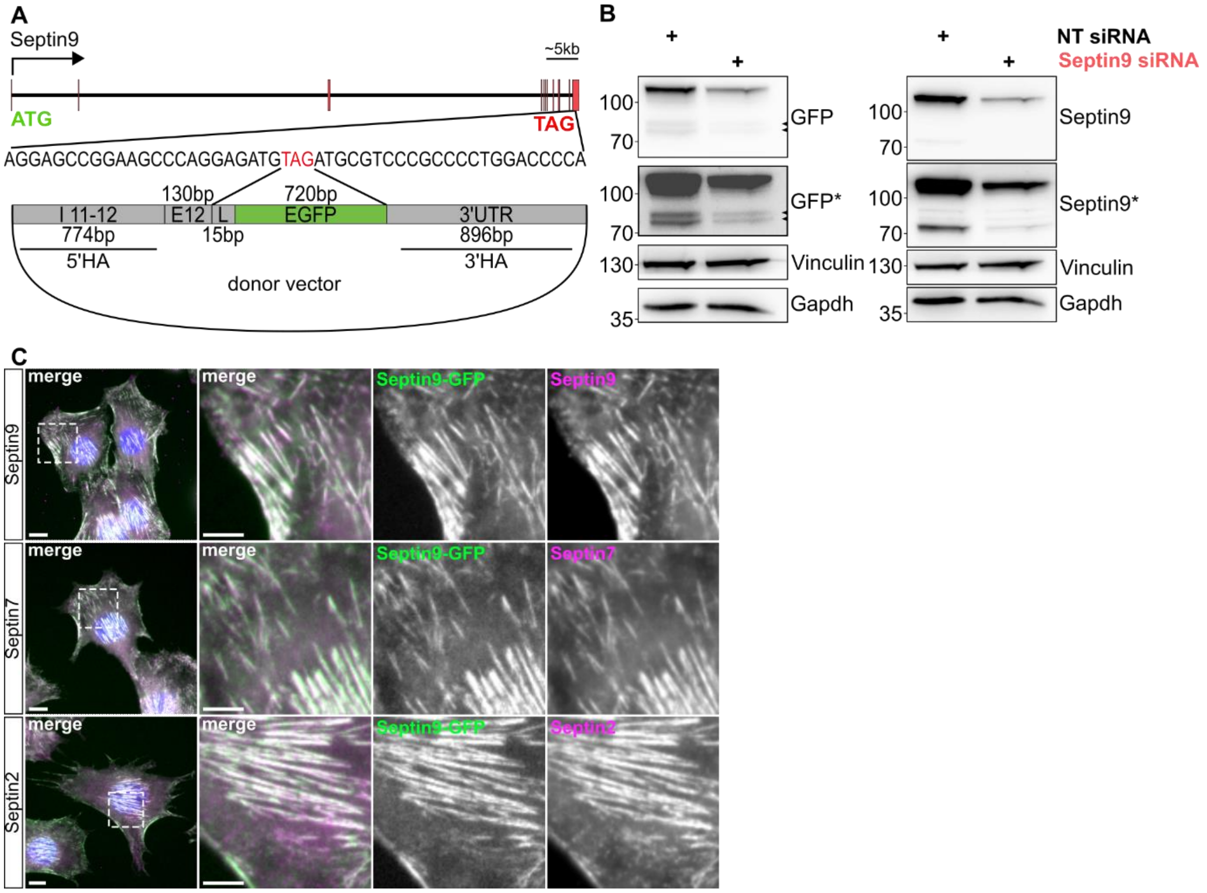

Figure S4 Generation and validation of a Septin9-GFP C2C12 cell line, Related to Figure 4.

A Cloning strategy introducing a 15bp linker (L) and an EGFP sequence instead of the stop codon of Septin9 in C2C12 cells. C-terminal strategy targets all Septin9 isoforms. Several clones were generated and tested. **B** Clone 32 was transfected with either non-targeting siRNA or Septin9 siRNA and subjected to a SDS PAGE analysis followed by a western blot. Antibodies against long Septin9 isoforms (run above 70kDa) and GFP were used to validate the knock-in. The molecular weight shift of about 30kDa (GFP) is visible with both antibodies. Probing the membrane with anti-GFP antibody might reveal the expression of the short isoforms (black arrow heads). Lanes marked with an asterisk (GFP* and Septin9*) represent longer exposure. **C** Representative epifluorescent images of proliferating Septin9-GFP C2C12 cells (clone#32) visualizing co-localization of Septin9-GFP with endogenous septin structures. Sepin9-GFP shows knock-in GFP signal, Septin9, Septin7 and Septin2 are stained with antibodies. Scale bar 10 µm, insets 5 µm.

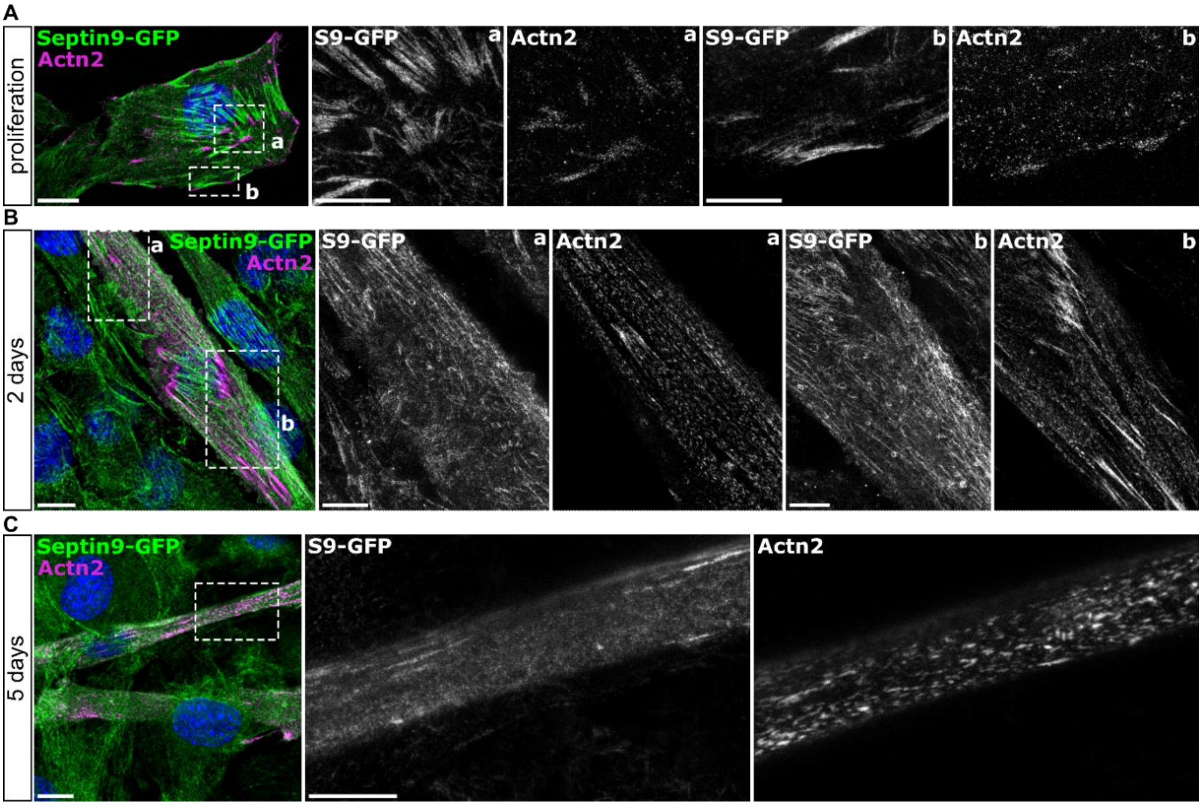

Figure S5 Septin9-GFP does not colocalize with α-actinin-2 during *in vitro* myogenesis. Related to Figure 4.

A Confocal (merge) and STED (insets) fluorescent micrographs of proliferating Septin9-GFP knock-in myoblasts. α-actinin-2 localizes to the focal adhesions and Septin9-GFP is excluded from these structures. **B** Representative images capture cytoskeletal reorganization in the nascent myotube after 2 days of differentiation. Septin9-GFP is no longer organized in long, straight filaments, but in short rods and rings, showing no colocalization with α-actinin-2. **C** The mature myotube shows no colocalization between residual Septin9-GFP and α-actinin-2, that resembles primitive sarcomeres. Scale bars 10 µm, insets in cells 5 µm.

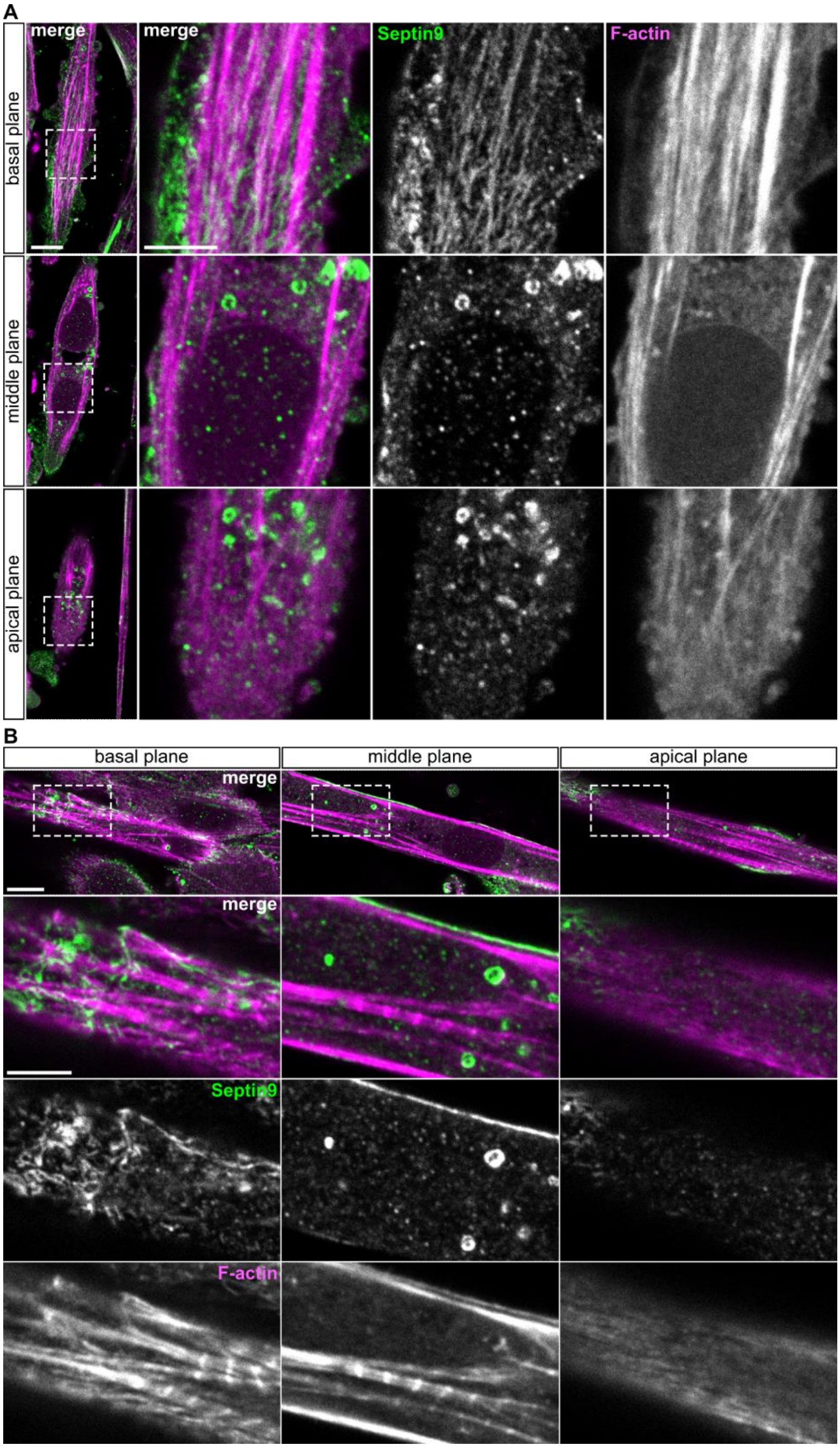

Figure S6 Sub-cellular distribution of native Septin9 in a myotube, Related to Figure 4.

Confocal images showing sub-cellular distribution of endogenous Septin9 after prolonged differentiation for 5 days. **A** Representative confocal images of a nascent myotube without apparent sarcomeric structures visualized with phalloidin. Increments in 6µm steps at the bottom, middle (6µm) and upper (12µm) plane of the wild type C2C12 cells. **B** Representative confocal images of a mature myotube with sarcomeres visualized with phalloidin. The bottom, middle (5µm) and upper (11µm) plane in of the wild type C2C12 cells. Magnified insets show Septin9 and Actin (phalloidin) organization in the boxed area of the myotube. Scale bar 10 µm, inset 5 µm.

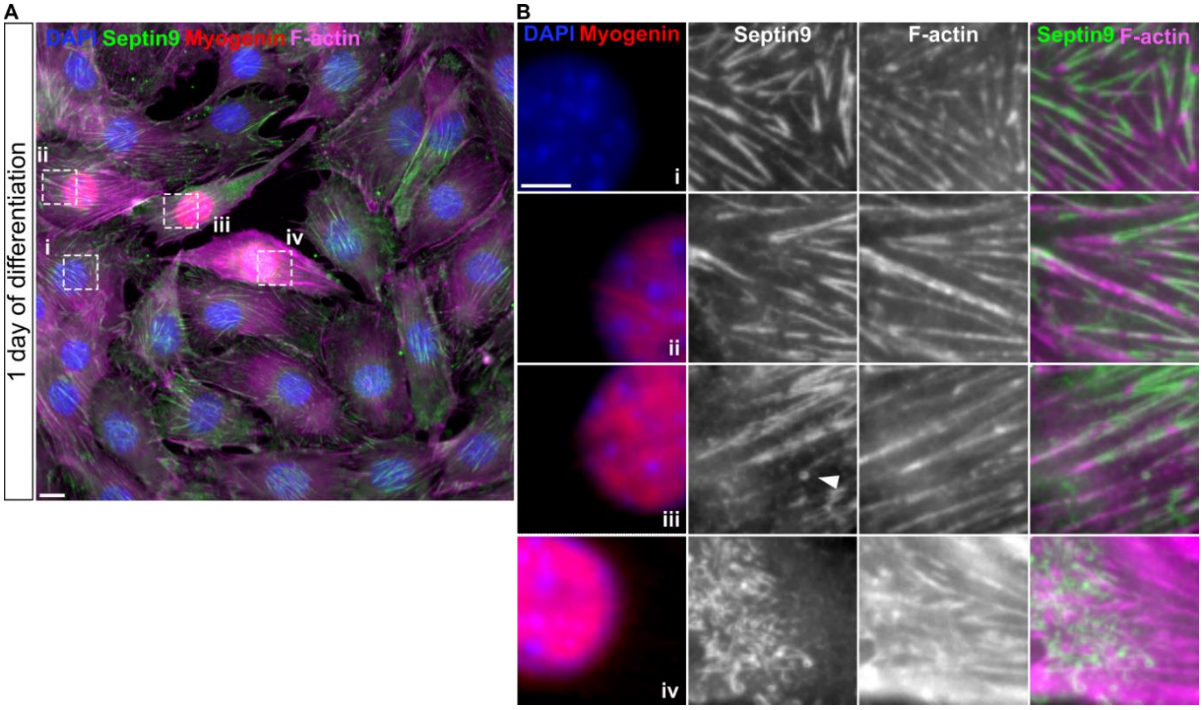

Figure S7 Septin9 reorganization is subsequent to Myogenin expression, Related to Figure 4.

A Representative epifluorescence image of wild type C2C12 cells differentiated for 1 day, visualizing Septin9, Myogenin and F-Actin via phalloidin. **B** Magnified insets from A show Septin9 organization in Myogenin-negative (inset i) and Myogenin positive cells (insets ii-iv). Myogenin-positive cells that display increased actin levels (inset iv) show the strongest Septin9 reorganization. White arrowhead shows a septin ring. Scale bar 10 µm, inset 5 µm.

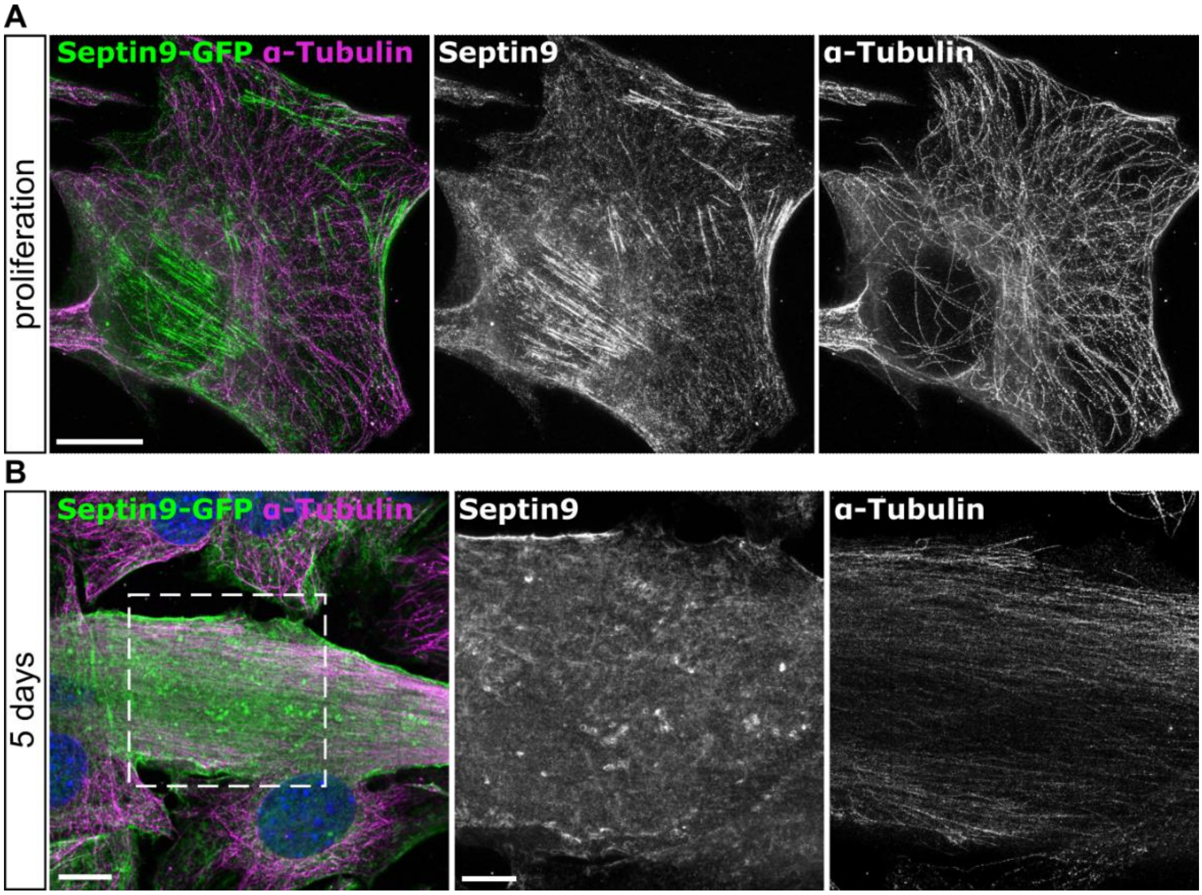

Figure S8 Microtubules in C2C12 cells are mostly devoid of Septin9, Related to Figure 4.

A Confocal (merge) and STED (insets) fluorescent micrographs of proliferating and differentiating for 5 days **B** Septin9-GFP knock-in myoblasts. Microtubules are visualized using α-Tubulin antibody. Septin9 appears to colocalize only occasionally at the cell periphery. Scale bars 10 µm, insets in cells 5 µm.

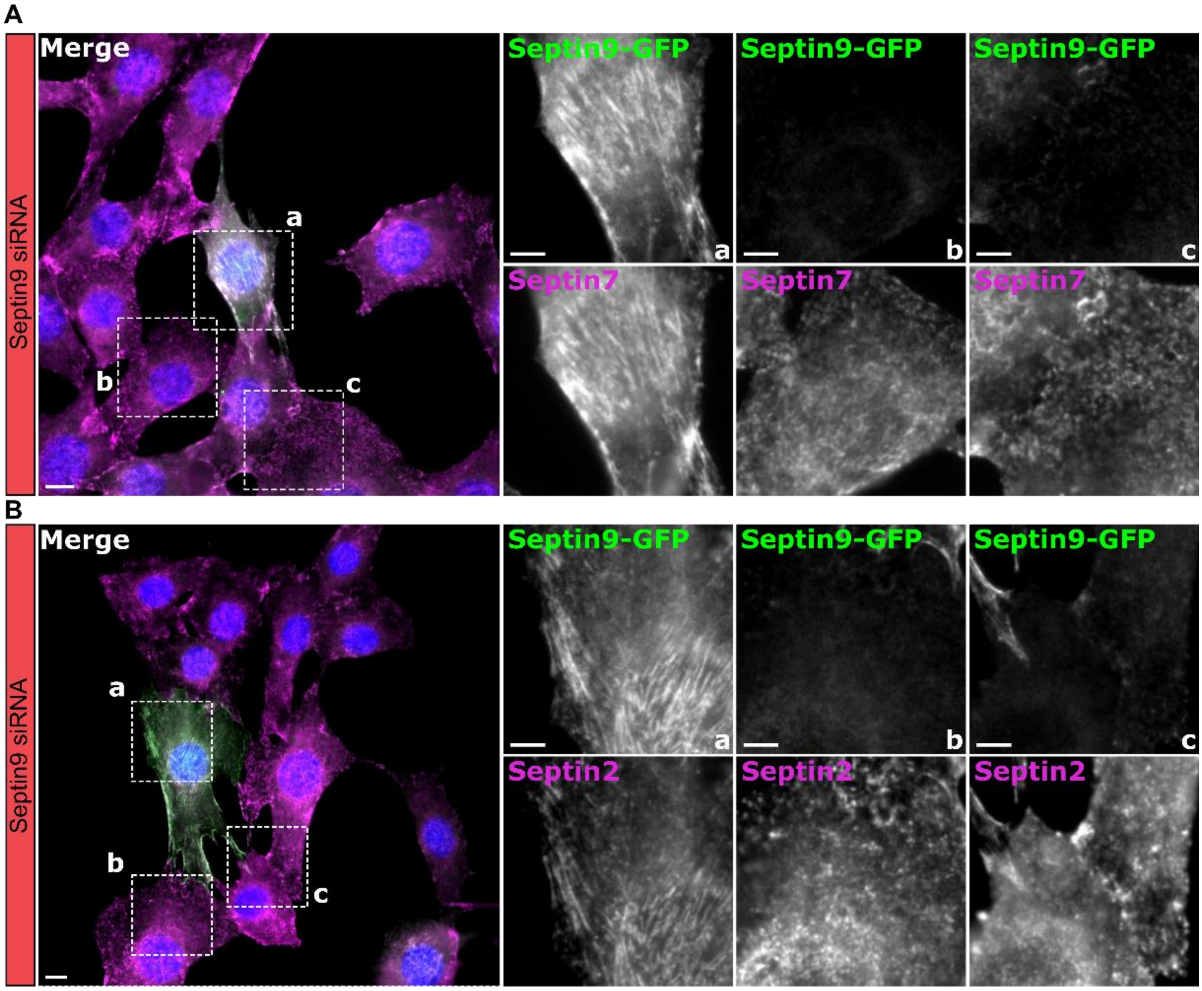

Figure S9 Integrity of septin filaments in absence of Septin9 in Septin9-GFP C2C12 cell line, Related to Figure 3.

A-B Representative epifluorescent images of proliferating Septin9-GFP clone#32 48 hours after siSeptin9 treatment visualizing Septin7 (**A**) and Septin2 (**B**). Sepin9-GFP shows knock-in GFP signal, Septin7 and Septin2 are stained with antibodies. Scale bar 10 µm, insets 5 µm.

Supplementary Tables

Table S1 Primers for RT-qPCR, related to STAR Methods

	Forward	Reverse
Myog	CCAAGGTCTCCTGTGCTGATG	TTGGCAAAACCACACAATGC
Myh2	GGAGGCTGAGGAACAATCCA	GTCATTCCACAGCATCGGGA
Myh8	AATGATGTTTCACAGCTGCAGAG	CCATCATGGCGGCATCAGTA
Rna18s1	CGGCTACCACATCCAAGGAA	GCTGGAATTACCGCGGCT
Septin2	GCCTGTCATTGCGAAAGCTG	GGGATACTGGCCTTGAGGAG
Septin4	CATCGTGGAAGTGGAAAACCC	GACAGCAGGGATAGGGAAGTC
Septin5	CAAGCAGTACGTTGGCTTCG	GTTGATGCGTTCCTCAGCAC
Septin6	CCCGACAGGACATTCGCTTA	AGGTGGGCATTCATGGTTCC
Septin7	GCCAACCTCCCAAATCAAGTG	ACTTTGGATTGCTCCACCTGT
Septin8	AGAGCGAGCTCCACAAGTTC	CAAAAGGCAGGTGTGCGTTC
Septin9	GGCTATGTGGGGATCGACTC	CGGCTGATTTTGGACTTGAA
Septin10	GAGATAAAAGAGCATCCCCGC	AATTCCAGTCTCCCCCACAC
Septin11	AGCCAGTGTCGGTAAAGAGC	GTTTCGCAGCTCTTCATTAC
Wwtr1	TCCCCACAACTCCAGAAGAC	CAAAGTCCCGAGGTCAACAT
Fst	CTCTTCAAGTGGATGATTTTC	ACAGTAGGCATTATTGGTCTG

Supplemental References

[1] Oprescu, S.N., Yue, F., Qiu, J., Brito, L.F., and Kuang, S. (2020). Temporal Dynamics and Heterogeneity of Cell Populations during Skeletal Muscle Regeneration. iScience *23*. https://doi.org/10.1016/j.isci.2020.100993.
